# Zr@IL-Fe_3_O_4_ MNPs as an efficient and green heterogeneous magnetic nanocatalyst for the one-pot three-component synthesis of highly substituted pyran derivatives under solvent-free conditions

**DOI:** 10.1039/d1ra04381a

**Published:** 2021-07-05

**Authors:** Mehraneh Aghaei-Hashjin, Asieh Yahyazadeh, Esmayeel Abbaspour-Gilandeh

**Affiliations:** Chemistry Department, University of Guilan 41335-1914 Rasht Iran yahyazadehphd@yahoo.com; Department of Chemistry, College of Science, University of Mohaghegh Ardabili Ardabil Iran abbaspour1365@yahoo.com

## Abstract

The present study was conducted to synthesize Zr@IL-Fe_3_O_4_ MNPs as a new magnetically recoverable heterogeneous catalyst, which was then characterized by Fourier transform infrared (FT-IR) spectroscopy, energy dispersive X-ray spectroscopy (EDX), vibrating sample magnetometry (VSM), X-ray diffraction (XRD), thermogravimetric analysis (TGA), scanning electron microscopy (SEM), and transmission electron microscopy (TEM) techniques. The catalytic behavior of the Zr@IL-Fe_3_O_4_ MNPs was efficiently used for the synthesis of highly substituted pyran derivatives *via* a one-pot three-component condensation of 4-hydroxycoumarin/dimedone, malononitrile, and arylaldehydes under solvent-free conditions. This new methodology demonstrated some important features, including short reaction times, excellent yields, lower loading of the catalyst, easy work-up, and recyclability of the catalyst for a minimum of six times without any noticeable decrease in catalytic activity.

## Abstract 2

Synthesis of highly substituted pyran derivatives using Zr@IL-Fe_3_O_4_ MNPs.

## Graphical abstract

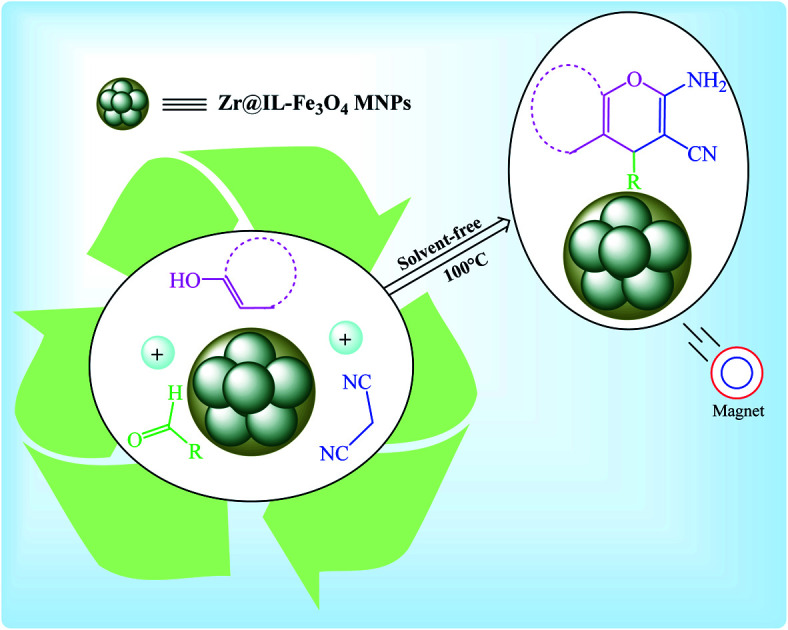

## Introduction

Magnetite (Fe_3_O_4_) core–shell nanocomposites are the most extensively surveyed magnetic nanoparticles (MNPs) and have been utilized for various applications, including medical diagnosis, color imaging, information storage, catalysis, and microwave absorption.^1–5^ MNPs, as a significant type of separable material, have recently attracted a great deal of interest among researchers for the synthesis of organic compounds and in materials science owing to their high surface area, high stability, and low toxicity.^6–8^ The high chemical reactivity and large surface area to volume ratio of magnetic nanoparticles has made them highly sensitive to oxidation and accumulation, respectively. One of the basic ways to overcome these problems and also to achieve further functionalization is to coat the surface of MNPs with organic or inorganic supports.^9,10^ Recently, efficient catalytic systems have been used as functionalized MNPs in several chemical developments.^11–20^ With regard to the high value of the magnetic catalysts employed in organic transformations and the precious materials synthesized by them, the simple recovery of the catalysts because of their strong magnetic fields and the capability to reuse them several times with a negligible decrease in their magnetic nature are two salient features in catalytic processes.^21,22^

Chromenes are interesting oxygen-containing heterocyclic molecules with a wide range of biological and pharmaceutical properties, such as antitubercular,^23^ molluscicidal,^24^ antifungal,^25^ antiproliferative, and antitumor^26^ activities. In addition, chromenes act as acetylcholinesterase inhibitors,^27^ as antagonists against antiapoptotic Bcl-2 protein,^28^ and as Src kinase inhibitors.^29^ Moreover, some of these compounds, as cognitive enhancers, can be employed for the treatment of neurodegenerative diseases, including Alzheimer's and Parkinson's diseases.^30^ Pyrano[3,2-*c*]coumarins are derivatives of chromenes, which have drawn attention of the scientific community over the last decades. The most important route for their preparation is the one-pot three-component condensation of 4-hydroxycoumarin with aldehyde and malononitrile in the presence of a catalyst. Several strategies for the construction of these types of coumarins have been reported under various conditions, such as H_6_P_2_W_18_O_62_·18H_2_O,^31^ nanoparticles,^32^ MgO,^33^ nanosilica,^34^ hexadecyltrimethyl ammonium bromide,^35^ ionic liquids,^36^ Mg/La mixed metal oxides,^37^ and (DAHP).^38^ Also, 4*H-*benzo-[*b*]-pyrans are other derivatives of heterocycles with a common structural motif in a diversity of natural and synthetic products. The acceleration of the synthesis of these compounds *via* the condensation reactions of dimedone, malononitrile, and aldehyde has been accomplished using numerous homogeneous or heterogeneous catalysts; for example, [DMImd-DMP],^39^ KNaC_4_H_4_O_6_·4H_2_O,^40^ K_2_CO_3_/cyanuric acid,^41^ nano-kaoline/BF_3_/Fe_3_O_4_,^42^ TMGT,^43^ and [γ-Fe_2_O_3_@Hap-Si-(CH_2_)_3_-AMP].^44^ Even though most of these methods offer distinct advantages, some of them have one or more limitations, such as low yields of the desired product, generation of a large amount of waste, long reaction times, poor recovery of the catalyst, and hard reaction conditions. Therefore, to avoid these limitations based on green chemistry protocols, the discovery of efficient, simple, versatile, and environmentally friendly processes for the preparation of highly substituted pyran derivatives is still favored.

## Experimental

All the pure chemical substances were obtained from Merck, Fluka, and Aldrich, chemical companies. The melting points of the heterocyclic compounds were recorded on an Electrothermal-9100 apparatus. Fourier transform infrared (FT-IR) spectra were recorded on a PerkinElmer PXI spectrometer in the range of 400–4000 cm^−1^ on KBr wafers. Magnetic susceptibility measurements by vibrating sample magnetometry were taken on a VSM system (MDK Co. Kashan, Iran), in the magnetic field range of −15 000–15 000 Oe at room temperature. X-ray diffraction (XRD) patterns of the samples were measured using a Philips-pw1730 system in the 2*θ* range of 10°–80° with Cu-Kα radiation (*λ* = 1.54 Å). Thermogravimetric analysis was recorded on a Linseis SATPT 100 thermoanalyzer under a N_2_ atmosphere at a heating rate of 10 °C min^−1^ over a temperature range of 25 °C–650 °C. Energy dispersive X-ray spectroscopy (EDX) of the as-prepared magnetic nanoparticles was performed on an FE-SEM (MIRA III, Detector from SAMX, France). Field emission electron microscopy (FESEM) images were obtained to survey the catalyst morphology utilizing an SEM-LEO 1430VP instrument. Transmission electron microscopy (TEM) images were recorded using a Zeiss-EM 900 instrument.

### General procedure for synthesis of dihydropyrano[3,2-*c*]chromene derivatives

A mixture of 4-hydroxycoumarin (1 mmol), malononitrile (1.2 mmol), aldehyde (1 mmol), and Zr@IL-Fe_3_O_4_ MNPs (20 mg) was stirred and heated in an oil-bath under solvent-free conditions. At the end of the reaction, as monitored by TLC (eluent: EtOAc : *n*-hexane), the catalyst was separated with an external magnetic field and the residue solid was washed with distilled water and recrystallized *via* EtOH in order to obtain the pure product 4.

### General procedure for synthesis of the 4*H-*benzo-[*b*]-pyran derivatives

A combination of dimedone (1 mmol), malononitrile (1.2 mmol), aldehyde (1 mmol), and Zr@IL-Fe_3_O_4_ MNPs (20 mg) was stirred and heated in an oil-bath under solvent-free conditions. At the end of the reaction, as monitored by TLC (eluent: EtOAc : *n*-hexane), the catalyst was separated using an external magnetic field and the residue solid was washed with distilled water and recrystallized *via* EtOH to obtain the pure product 6.

### Preparation of the Fe_3_O_4_ MNPs

Primarily, a mixture containing 0.86 g FeCl_2_·4H_2_O and 2.36 g FeCl_3_·6H_2_O was added to the 40 mL deionized water under argon flow. The mixture was then stirred at 90 °C under an argon atmosphere until the salts dissolved completely. Subsequently, 10 mL ammonia solution (25%) was added drop-wise to the reaction mixture and stirred at this temperature for another 20 min under an argon flow. The black precipitates were isolated with the help of a permanent magnet and rinsed with distilled water followed by being dried in an oven.

### Preparation of the Fe_3_O_4_@SiO_2_ (SCMNPs)

In a typical process, 60 mL ethanol, 20 mL deionized water, 2 mL ammonia solution (25%), and 1.0 g of the obtained Fe_3_O_4_ nanoparticles were placed into a 250 mL round-bottom flask and sonicated for 10 min. Afterwards, 0.45 mL tetraethylorthosilicate (TEOS) was added to the reaction solution and sonicated for another 10 min. The resulting dispersion was stirred for 14 h at ambient temperature and collected from the reaction solution with magnetic decantation. The dispersion was rinsed several times with a mixture of ethanol and water (1 : 1) and dried under vacuum.

### Preparation of the Amp@SCMNPs

First, 1 g SCMNPs was dispersed in 20 mL dry toluene with the aid of ultrasonication for 30 min and 2 mL 3-aminopropyltriethoxysilane (Amp) was added into the resulting solution. The resulting mixture was refluxed under vigorous stirring for 24 h under an argon atmosphere and separated with magnetic decantation in the presence of a permanent magnetic field. The precipitates were rinsed three times with water and ethanol and dried under a vacuum oven.

### Preparation of the ThAl/Amp@SCMNPs

First, 1 g of dispersed Amp@SCMNPs nanoparticles was added to a 30 mL hot methanolic solution containing 5 mmol of 2,5-thiophene-dicarboxaldehyde (ThAl) and stirred under reflux for 12 h in a water bath. The achieved precipitates (ThAl/Amp@SCMNPs) were isolated *via* magnetic decantation, rinsed with ethanol several times to eliminate the excess chemicals, and dried under a vacuum oven.

### Preparation of the IL/ThAl@SCMNPs

First, 1 g of the prepared ThAl/Amp@SCMNPs was added to the reaction solution containing 50 mL of EtOH and dispersed by sonication for 30 min. After this period of time, 5 mmol isoleucine was poured in to the reaction vessel and refluxed for 12 h under stirring. The residue precipitates (IL/ThAl@SCMNPs) were collected using a permanent magnetic field, rinsed three times with ethanol, and dried in a vacuum oven.

### Preparation of the Zr@IL-Fe_3_O_4_ MNPs

**Scheme 1:**
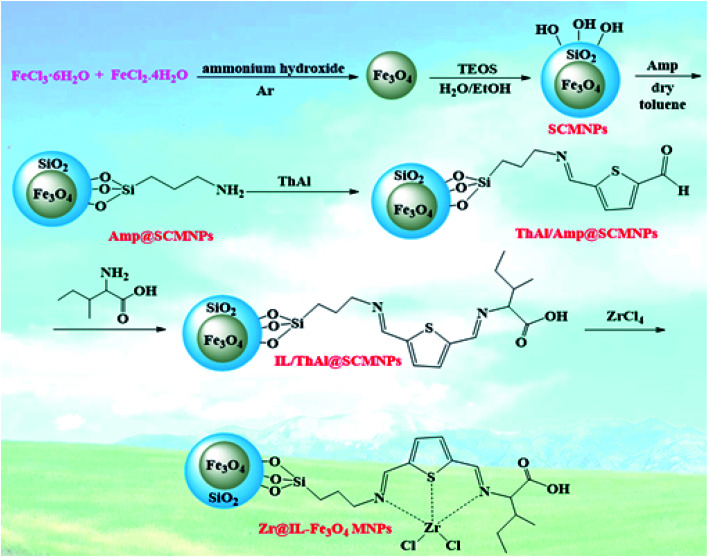
Synthesis of the Zr@IL-Fe_3_O_4_ MNPs.

First, 0.34 g ZrCl_4_ was added into the reaction solution containing 1 g of dispersed IL/ThAl@SCMNP nanoparticles in 80 mL ethanol and stirred vigorously for 12 h. Then, the precipitates were isolated by magnetic decantation in the presence of a permanent magnetic field. The residue solid (Zr@IL-Fe_3_O_4_ MNPs) was rinsed three times with water and ethanol and dried under a vacuum oven. Scheme 1 exhibits all the stages for the synthesized catalyst.

## Results and discussion

### FT-IR analysis

**Fig. 1:**
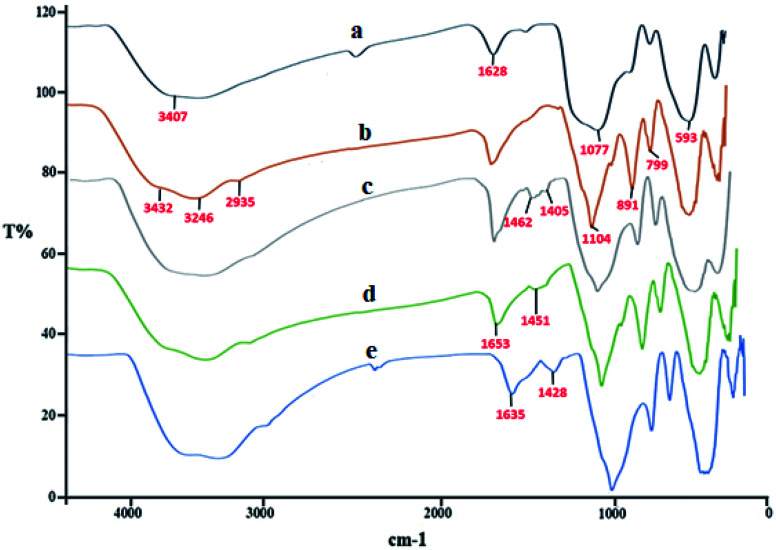
FT-IR spectra of SCMNPs (a), Amp@SCMNPs (b), ThAl/Amp@SCMNPs (c), IL/ThAl@SCMNPs (d), and Zr@IL-Fe_3_O_4_ MNPs (e).

Fig. 1 presents the FT-IR of SCMNPs (a), Amp@SCMNPs (b), ThAl/Amp@SCMNPs (c), IL/ThAl@SCMNPs (d), and Zr@IL-Fe_3_O_4_ MNPs (e). In the spectrum of SCMNPs, the characteristic peaks at 593 and 3407 cm^−1^ could be observed, which could be relevant to the stretching vibrations of Fe–O–Fe and O–H, respectively. The characteristic peaks at 1077 and 1628 cm^−1^ could be attributed to the stretching vibrations of Si–O–Si and twisting bonds of Si–O–H and H–O–H, respectively. The presence of C–H stretching modes, which were assigned to the 3-aminopropyltriethoxysilane in the FT-IR of Amp@SCMNPs, was confirmed by the stretching vibrations at about 2935 cm^−1^. Two peaks for asymmetric stretching (799 cm^−1^) and in-plane bending (891 cm^−1^) of the Si–O–Si group can be observed in the related spectrum. Moreover, the characteristic peaks at around 3246 and 3432 cm^−1^ were assigned to the stretching vibrations of NH_2_ groups, indicating the successful covalent attachment of Amp to the silica layer surface. The presence of 2,5-thiophene-dicarboxaldehyde in the FT-IR of ThAl/Amp@SCMNPs was confirmed with C=C stretching and ring stretching at about 1462 and 1405 cm^−1^, respectively. In the FT-IR spectrum of IL/ThAl@SCMNPs, the peaks for C–N (1451 cm^−1^) and COO (1653 cm^−1^) stretching vibrations could be observed. The characteristic peak at about 3000 to 3400 cm^−1^ was assigned to the acidic OH stretching vibrations, suggesting that the isoleucine was successfully attached to the ThAl/Amp@SCMNP surface. The FT-IR spectra of the Zr@IL-Fe_3_O_4_ MNPs showed a frequency shift for certain bonds, indicating the coordination of the zirconia with the desired bonds.

### EDX analysis

**Fig. 2:**
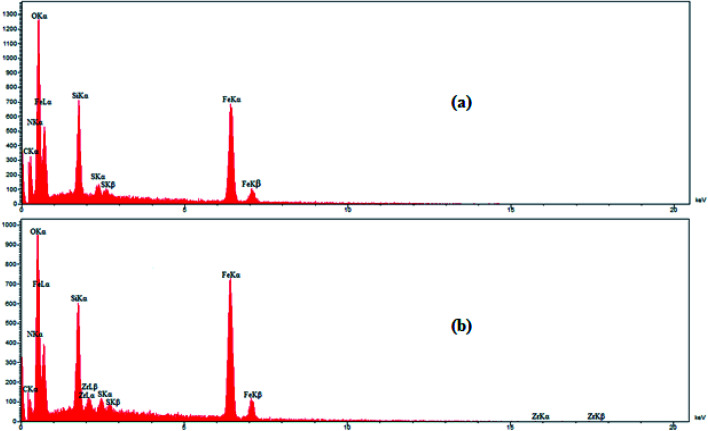
EDX spectra of IL/ThAl@SCMNPs (a) and Zr@IL-Fe_3_O_4_ MNPs (b).

**Fig. 3:**
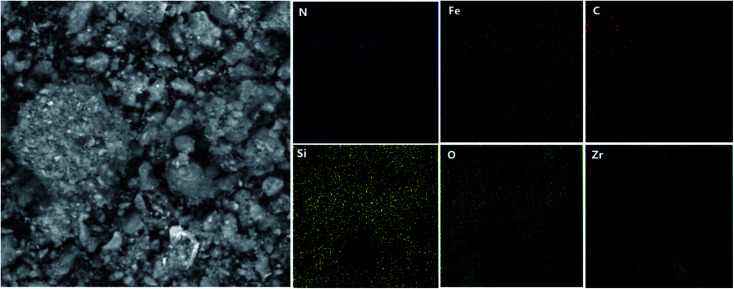
SEM image of the Zr@IL-Fe_3_O_4_ MNP nanocatalyst and the corresponding quantitative EDX element mapping of C, N, O, Fe, Si, and Zr.

Energy dispersive X-ray (EDX) analysis was employed to obtain information on the element distribution in the structure of IL/ThAl@SCMNPs (a) and Zr@IL-Fe_3_O_4_ MNPs (b) (Fig. 2). In the case of IL/ThAl@SCMNPs, the presence of C, N, O, Fe, Si, and S signals confirmed the loading of the functional groups on the surface of the magnetic nanoparticles. Based on the EDX analysis of the Zr@IL-Fe_3_O_4_ MNPs and the presence of the zirconia element, it could be concluded that the catalyst had been successfully synthesized. Also, the elemental map of the Zr@IL-Fe_3_O_4_ MNP nanocatalyst exhibited the presence of C, N, O, Fe, Si, and Zr elements (Fig. 3). Morever, the content of Zr in Zr@IL-Fe_3_O_4_ MNPs (4.6% of Zr anchored on the catalyst) was determined by inductively coupled plasma atomic emission spectrometry.

### VSM analysis

**Fig. 4:**
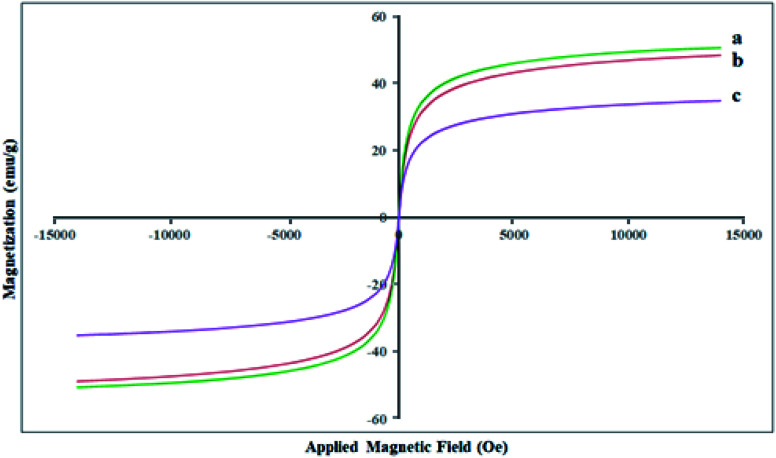
VSM magnetization curves of the Fe_3_O_4_ MNPs (a), SCMNPs (b), and Zr@IL-Fe_3_O_4_ MNPs (c).

The magnetic features of the Fe_3_O_4_ MNPs, SCMNPs, and Zr@IL-Fe_3_O_4_ MNPs were determined by vibrating sample magnetometry (VSM) at ambient temperature (Fig. 4). The saturation magnetization (Ms) quantity of the Fe_3_O_4_ MNPs was obtained as 50.63 emu g^−1^. For the SCMNPs, the quantity of the saturation magnetization was 48.40 emu g^−1^, which was lower than that of the Fe_3_O_4_ MNPs. Furthermore, the saturation magnetization quantity (Ms) was 34.83 for Zr@IL-Fe_3_O_4_ MNPs. These outcomes exhibited that the magnetization of the Fe_3_O_4_ MNPs decreased during the functionalization process with silica layers, organic molecules, and metal groups. Despite this decrease in the saturation magnetization quantity, Zr@IL-Fe_3_O_4_ MNPs could be separated from the reaction mixture using a conventional magnet.

### XRD analysis

**Fig. 5:**
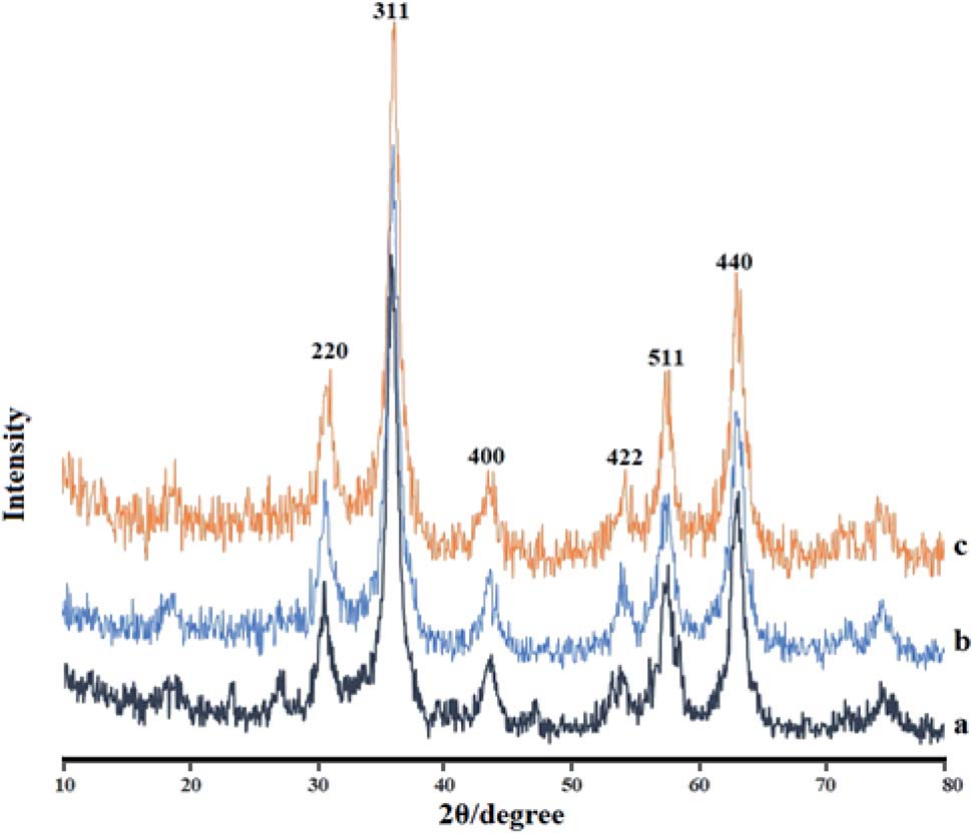
X-ray diffraction results for the Fe_3_O_4_ MNPs (a), IL/ThAl@SCMNPs (b), and Zr@IL-Fe_3_O_4_ MNPs (c).

Fig. 5 depicts the X-ray diffraction (XRD) patterns of the Fe_3_O_4_ MNPs (a), IL/ThAl@SCMNPs (b), and Zr@IL-Fe_3_O_4_ MNPs (c). In the XRD pattern of the Fe_3_O_4_ MNPs, the strong diffraction peaks at 2*θ* values, including at 30.55°, 35.74°, 43.81°, 53.65°, 57.52°, and 63.08°, were attributed to (220), (311), (400), (422), (511), and (440), respectively. The same sets of characteristics peaks were also observed for the IL/ThAl@SCMNPs and Zr@IL-Fe_3_O_4_ MNPs. This revealed that the crystalline structure of Fe_3_O_4_ MNPs did not lead to their phase change during surface modification.

### TGA analysis

**Fig. 6:**
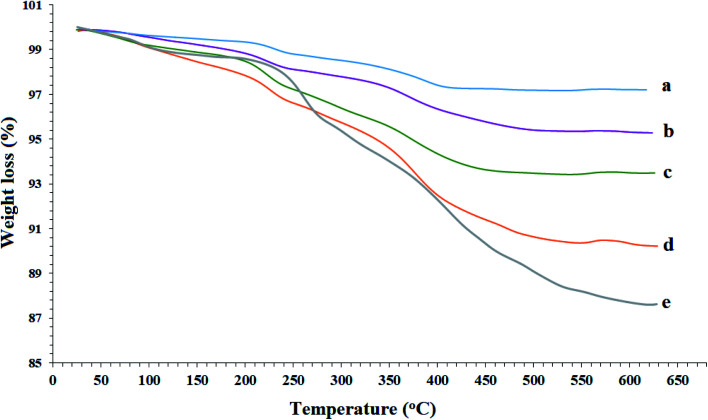
TGA curves of the Fe_3_O_4_ MNPs (a), SCMNPs (b), ThAl/Amp@SCMNPs (c), IL/ThAl@SCMNPs (d), and Zr@IL-Fe_3_O_4_ MNPs (e).

As shown in Fig. 6, the thermal stability of the Fe_3_O_4_ MNPs (a), SCMNPs (b), ThAl/Amp@SCMNPs (c), IL/ThAl@SCMNPs (d), and Zr@IL-Fe_3_O_4_ MNPs (e) were examined through thermogravimetric analysis (TGA) with a heating rate of 10 °C min^−1^ under a nitrogen stream. In all the samples, the weight loss below 250 °C was attributed to water thermodesorption from the surface (drying). The TGA graph of the SCMNPs exhibited a weight loss up to 650 °C due to decomposition of the hydroxyl ions on the surface of the magnetic nanoparticles and volatilization. In the TGA curves of the ThAl/Amp@SCMNPs and IL/ThAl@SCMNPs, another weight loss could be seen in the range between 335 °C to 440 °C, which could be attributed to the decomposition of the organic groups grafted on to the surface of the magnetic nanoparticles. Additionally, a two-step weight loss of the Zr@IL-Fe_3_O_4_ MNPs in the range between 340 °C to 560 °C could be ascribed to the decomposition of the organic parts and metal groups.

### TEM analysis

**Fig. 7:**
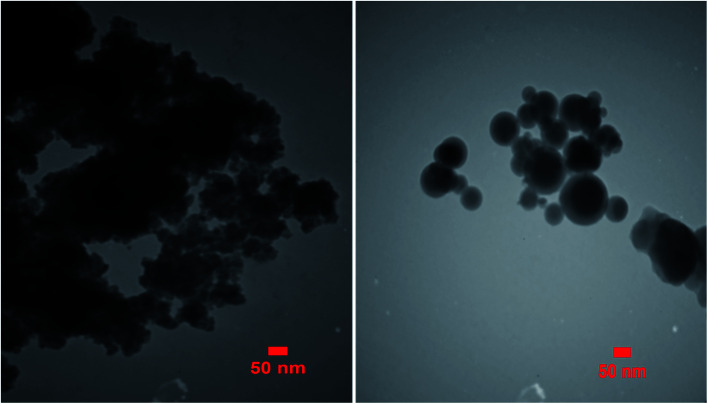
TEM image of the Zr@IL-Fe_3_O_4_ MNPs.

The TEM micrograph of the Zr@IL-Fe_3_O_4_ MNPs is demonstrated in Fig. 7. Based on this image, the obtained catalyst had a mean diameter of roughly 35 nm and the particles indicated a nearly spherical morphology with a narrow size distribution.

### SEM analysis

**Fig. 8:**
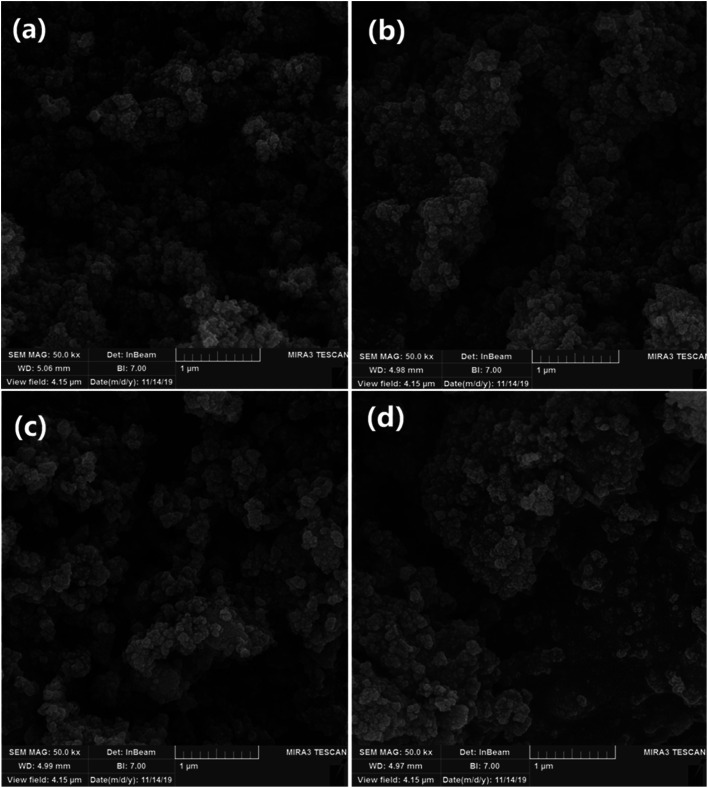
SEM images of the: Fe_3_O_4_ MNPs (a), SCMNPs (b), IL/ThAl@SCMNPs (c), and Zr@IL-Fe_3_O_4_ MNPs (d).

Fig. 8 shows the typical scanning electron microscopy (SEM) images of the Fe_3_O_4_ MNPs (a), SCMNPs (b), IL/ThAl@SCMNPs (c), and Zr@IL-Fe_3_O_4_ MNPs (d). As could be seen in the images of Fe_3_O_4_ and SCMNPs, the synthesized spherical particles had smooth surfaces and their average diameters were 19 and 26 nm, respectively. The SEM images of IL/ThAl@SCMNPs and Zr@IL-Fe_3_O_4_ MNPs exhibited that in the nanoparticles, no considerable changes could be observed after attachment of the organic parts or metal groups to the surface of the Fe_3_O_4_ MNPs and their average sizes ranged from 26 to 35 nm.

**Scheme 2:**
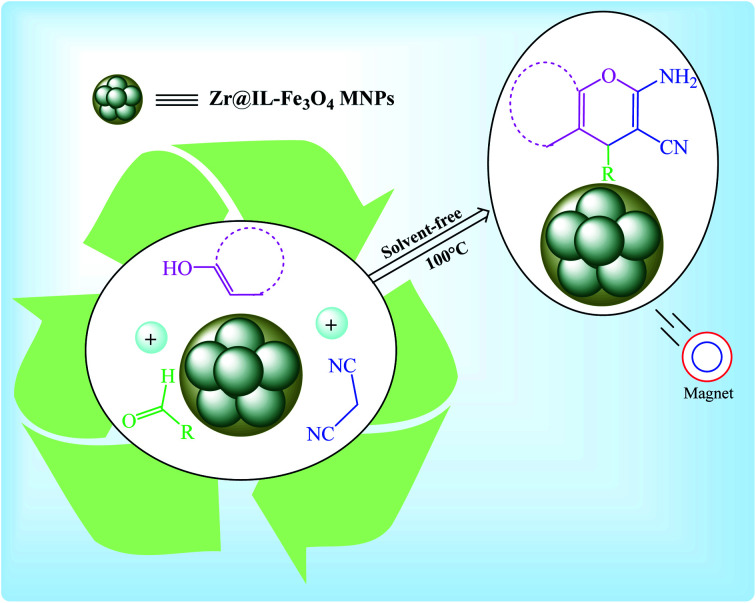
Synthesis of highly substituted pyran derivatives using the Zr@IL-Fe_3_O_4_ MNPs.

In this research, we report our outcomes for the preparation of highly substituted pyran derivatives using Zr@IL-Fe_3_O_4_ MNPs as a novel, effective, and reusable heterogeneous magnetic nanocatalyst under solvent-free conditions (Scheme 2).

To acquire the optimized reaction conditions for the preparation of polyfunctionalized dihydropyrano[3,2-*c*]chromene (4) derivatives, the reaction among 4-hydroxycoumarin (1 mmol), malononitrile (1.2 mmol), and benzaldehyde (1 mmol) was analyzed under various conditions, for instance, different temperatures, amounts of catalyst, and solvent (Table 1). To discover the effect of the solvent, the reaction performance was carried out with various solvents, such as CH_3_CN, H_2_O, EtOH, CH_2_Cl_2_, and toluene (Table 1, entries 1–5). The investigations into the selected solvents showed that in the presence of CH_3_CN, the desired product was achieved in good yields in short reaction times (Table 1, entry 1). Once the model reaction was applied in the rest of the above-mentioned solvents, the reaction proceeded rapidly for producing the target product (4a) in excellent yields (Table 1, entry 6). In our study, the influence of the catalyst concentration on the model reaction was also surveyed at 10, 15, 20, and 25 mg to examine their performance under solvent-free conditions (Table 1, entries 6 and 7–9). Among the tested concentrations under solvent-free conditions, the best outcome belonged to the existence of 20 mg of Zr@IL-Fe_3_O_4_ MNPs (Table 1, entry 6). Any decrease or increase in the concentration of the catalyst did not improve the reaction times or product yields (Table 1, entries 7–9). To illustrate the effect of temperature on the completion of the reaction in the presence of 20 mg of catalyst under solvent-free conditions, various temperatures from 25 °C to 120 °C were evaluated (Table 1, entries 6 and 10–16). As shown in Table 1, using 20 mg of the Zr@IL-Fe_3_O_4_ MNPs under solvent-free conditions at 100 °C resulted in the highest yield of the desired product in a short reaction time (Table 1, entry 6). It is noteworthy that when the model reaction was performed at a temperature below 100 °C, low-to-high yields of the product were achieved (Table 1, entries 9–14). It was also found that at a higher temperature than 100 °C, the reaction yield could be obtained at relatively low values (Table 1, entries 15–16).

**Table 1:** Effect of the solvent, amount of the catalyst, and temperature on the three-component reaction of 4-hydroxycoumarin (1), malononitrile (2), and benzaldehyde (3a), under various conditions

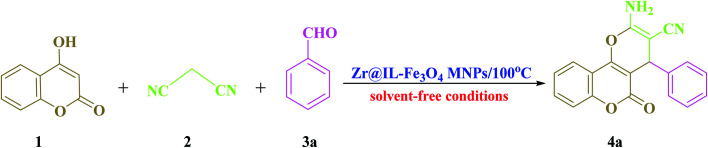					
Entry	Solvent	Catalyst (mg)	Temp.	Time (min)	Yield^a^ (%)
1	CH_3_CN	20	Reflux	60	73
2	H_2_O	20	Reflux	60	58
3	EtOH	20	Reflux	60	69
4	CH_2_Cl_2_	20	Reflux	60	44
5	Toluene	20	Reflux	60	53
6	—	20	100 °C	10	96
7	—	10	100 °C	35	74
8	—	15	100 °C	20	85
9	—	25	100 °C	10	94
10	—	20	25 °C	90	41
11	—	20	60 °C	55	62
12	—	20	70 °C	35	73
13	—	20	80 °C	25	86
14	—	20	90 °C	20	92
15	—	20	110 °C	10	95
16	—	20	120 °C	15	91

a The yields refer to the isolated product.

In order to popularize the optimum conditions and determine the priority and the acceptability of the method, various derivatives of dihydropyrano[3,2-*c*]chromene (4a–q) were achieved with high purity in high-to-excellent yields from the one-pot condensation of 4-hydroxycoumarin (1), malononitrile (2), and a wide range of aromatic aldehydes containing electron-withdrawing as well as electron-donating groups (3) in the presence of Zr@IL-Fe_3_O_4_ MNPs under solvent-free conditions (Table 2).

**Table 2:** Catalytic activity of the Zr@IL-Fe_3_O_4_ MNPs in the synthesis of dihydropyrano[3,2-*c*]chromenes under solvent-free conditions^a^

Entry	Aldehyde	Time (min)	Yield^b^ (%)	M.P. (obsd) (°C)	M.P. (lit.) (°C)	Product
1	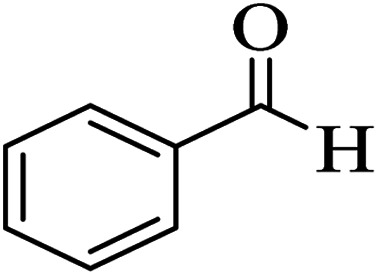	10	96	254–256	255–256 (ref. 45)	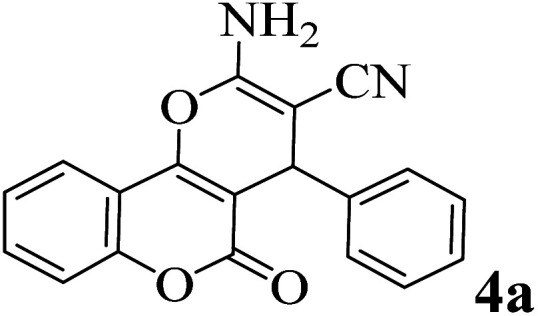
2	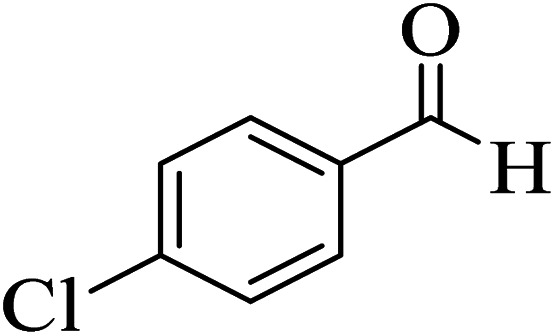	15	96	257–259	258–260 (ref. 46)	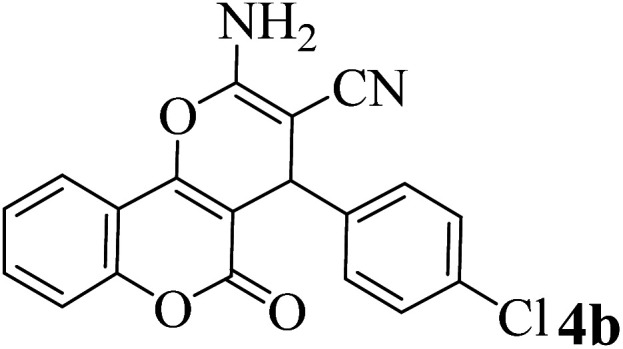
3	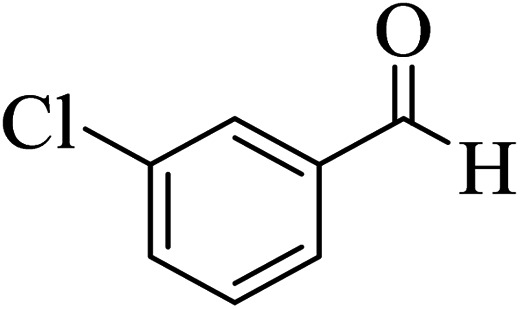	15	92	247–249	246–248 (ref. 47)	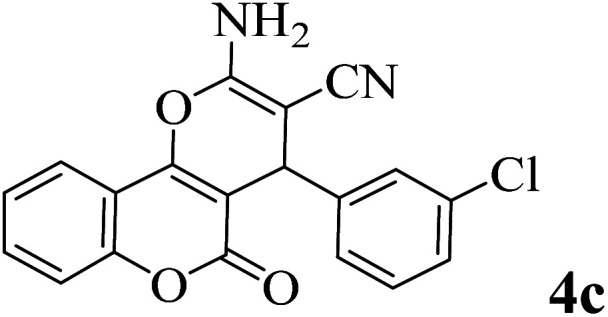
4	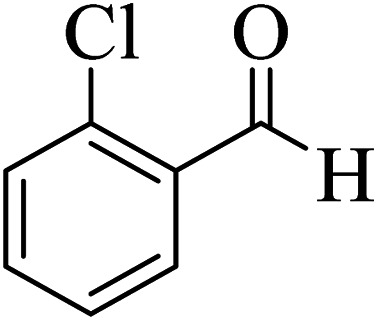	15	95	267–269	266–268 (ref. 48)	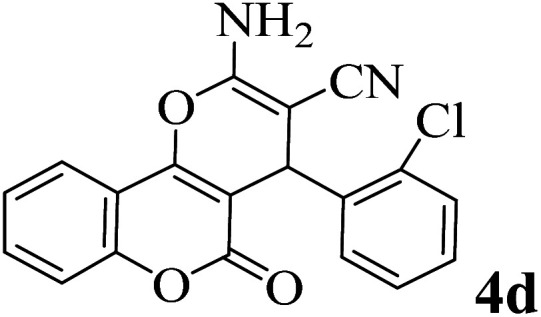
5	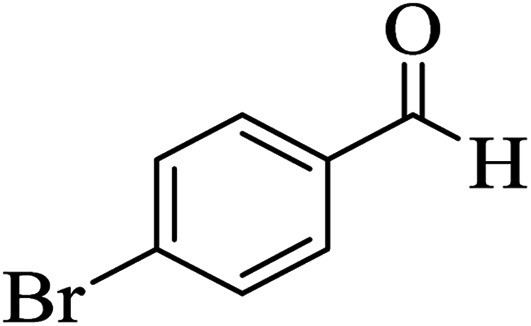	15	92	248–251	252–254 (ref. 49)	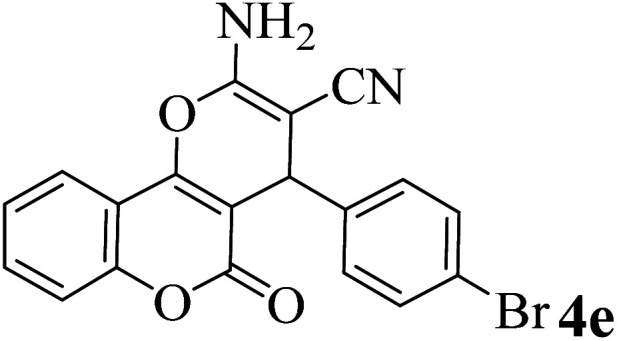
6	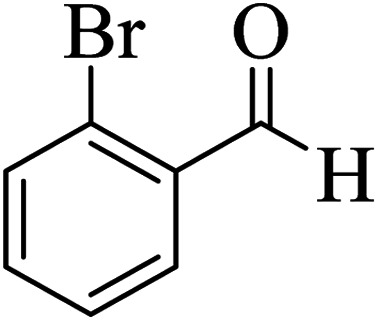	20	94	290–292	295–297 (ref. 48)	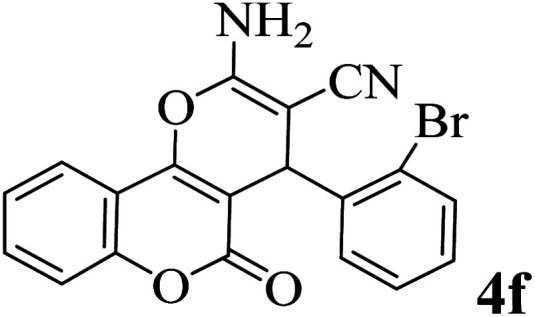
7	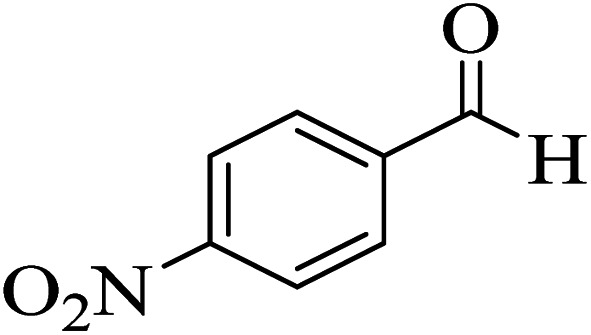	15	92	255–257	258–260 (ref. 38)	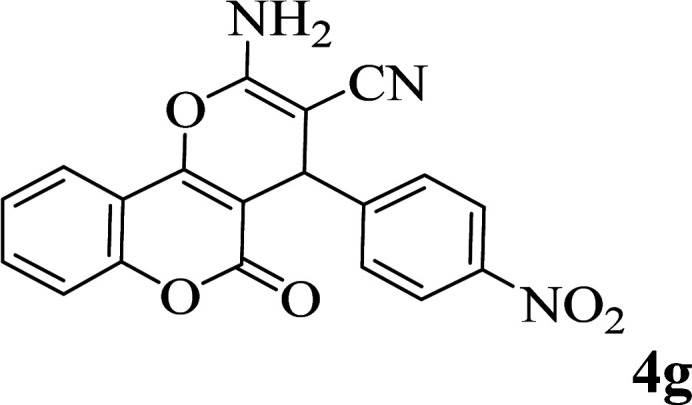
8	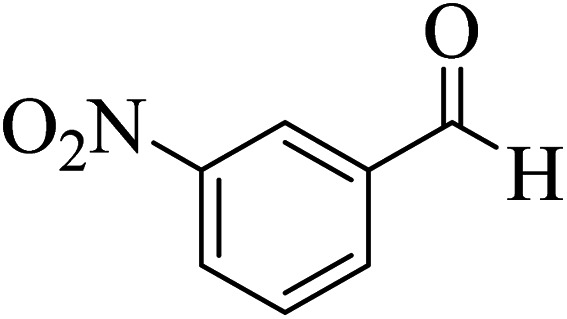	15	94	258–260	261–262 (ref. 45)	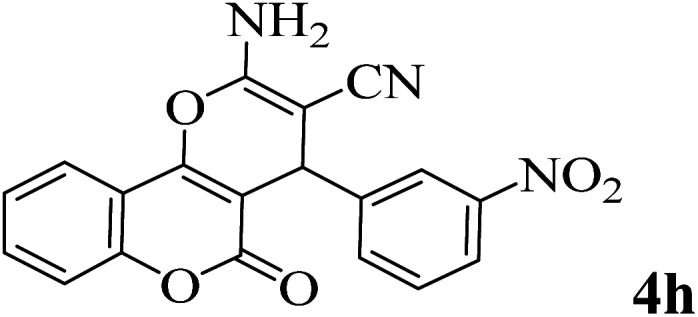
9	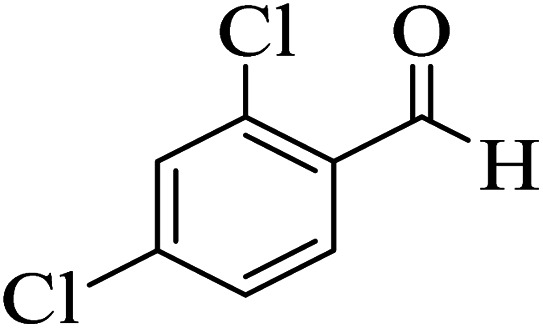	15	96	254–256	257–259 (ref. 38)	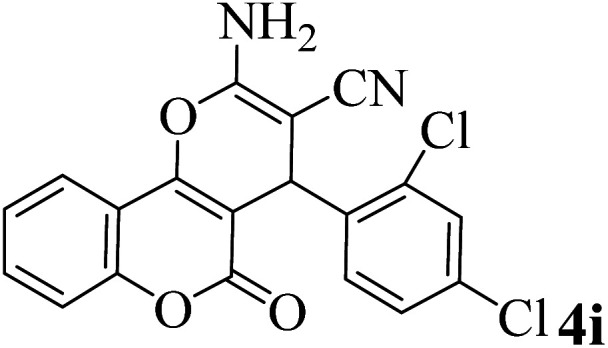
10	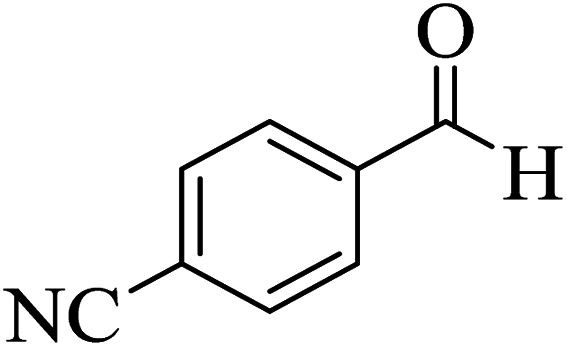	15	92	285–287	289–290 (ref. 48)	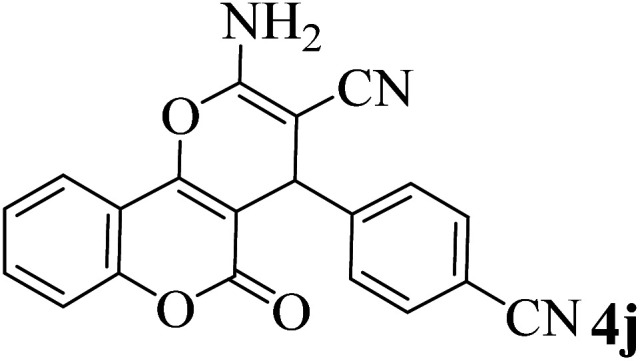
11	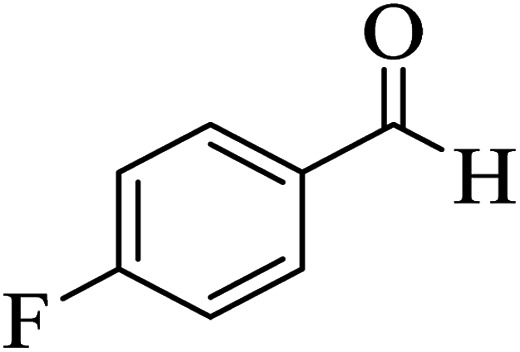	15	95	259–261	260–262 (ref. 47)	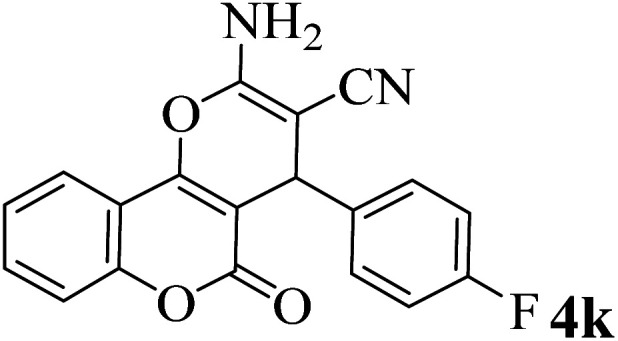
12	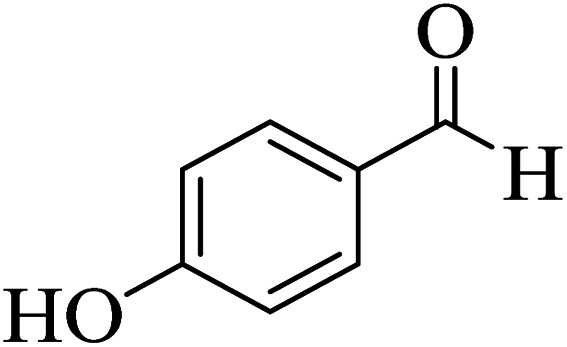	20	92	263–265	266–267 (ref. 49)	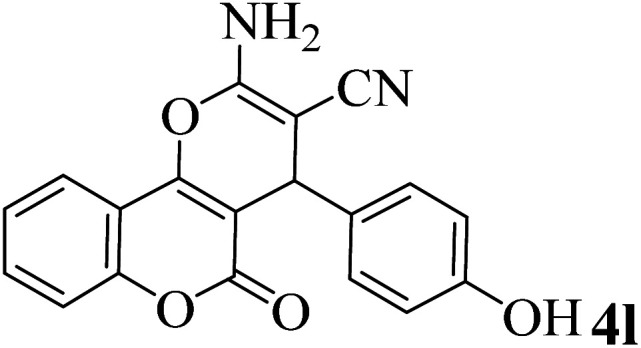
13	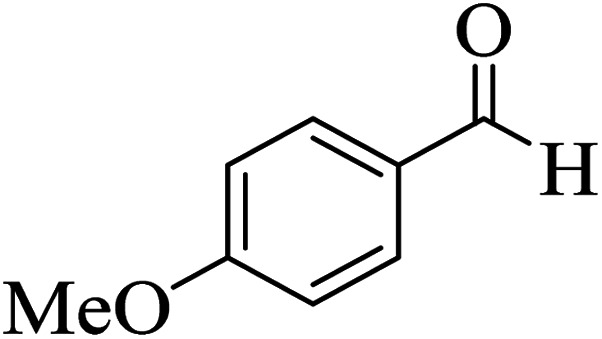	25	90	225–227	222–224 (ref. 45)	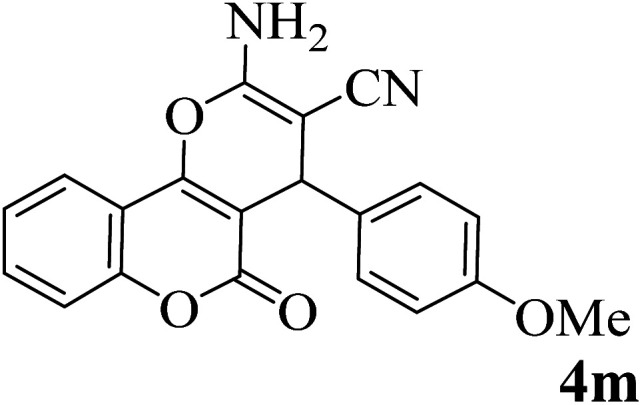
14	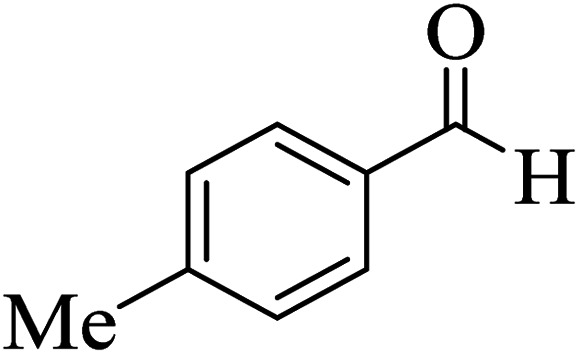	25	91	252–254	251–253 (ref. 47)	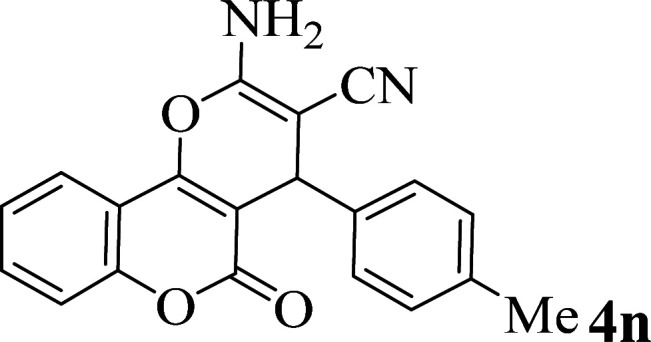
15	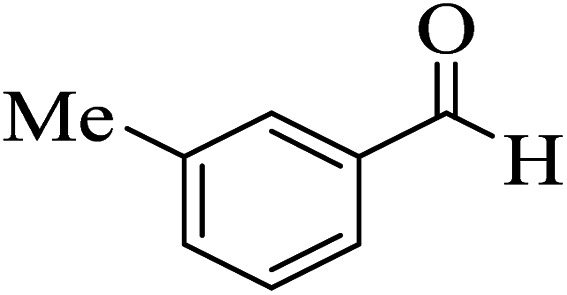	25	91	253–255	250–252 (ref. 50)	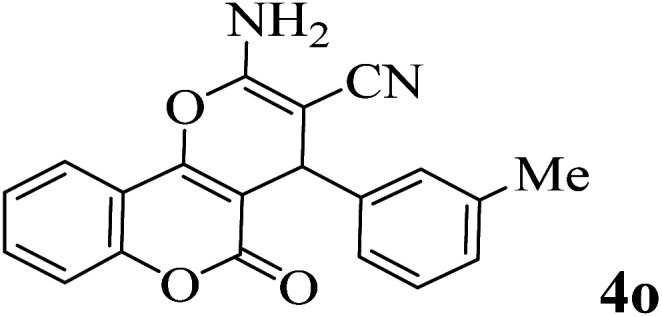
16	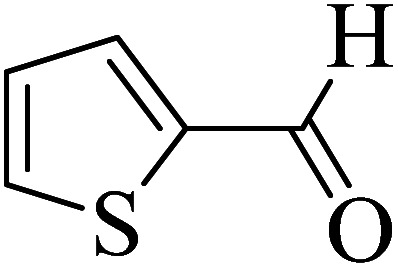	25	91	224–226	227–229 (ref. 51)	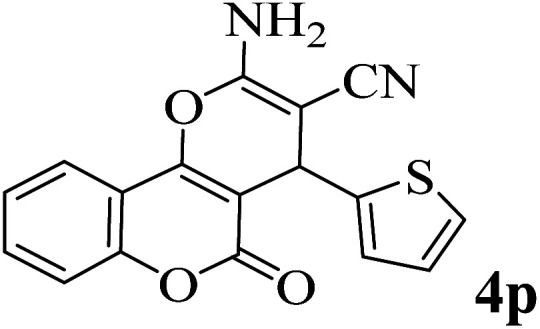
17	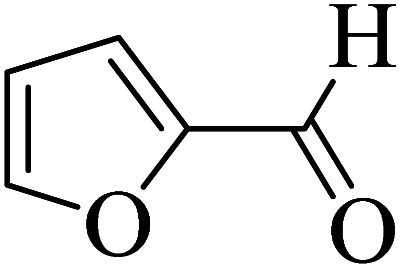	25	91	252–254	252–253 (ref. 52)	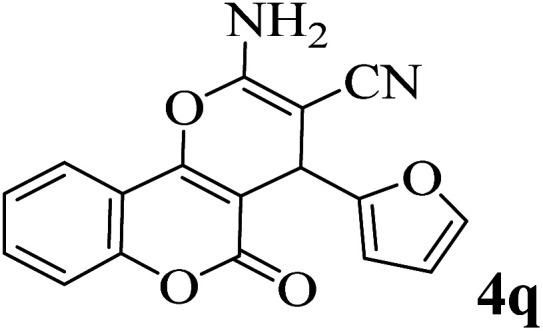

a Reaction conditions: 4-hydroxycoumarin (1 mmol), malononitrile (1.2 mmol), aldehyde (1 mmol), Zr@IL-Fe_3_O_4_ MNPs (20 mg), solvent-free.

b The yields refer to the isolated product.

In addition, to expand the use of the prepared magnetic nanocatalyst for other reactions related to this classification, a series of polyfunctionalized 4*H-*benzo-[*b*]-pyran complexes (6a–n) were prepared from the reaction mixture of dimedone (5), malononitrile (2), and the aryl aldehydes (3) under the optimized reaction conditions mentioned above (Table 3), which is described in the following:

**Table 3:** Catalytic activity of the Zr@IL-Fe_3_O_4_ MNPs in the synthesis of 4*H-*benzo-[*b*]-pyrans under solvent-free conditions^a^

Entry	Aldehyde	Time (min)	Yield^b^ (%)	M.P. (obsd) (°C)	M.P. (lit.) (°C)	Product
1	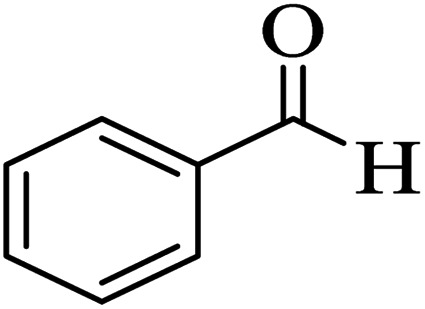	17	91	233–235	231–233 (ref. 53)	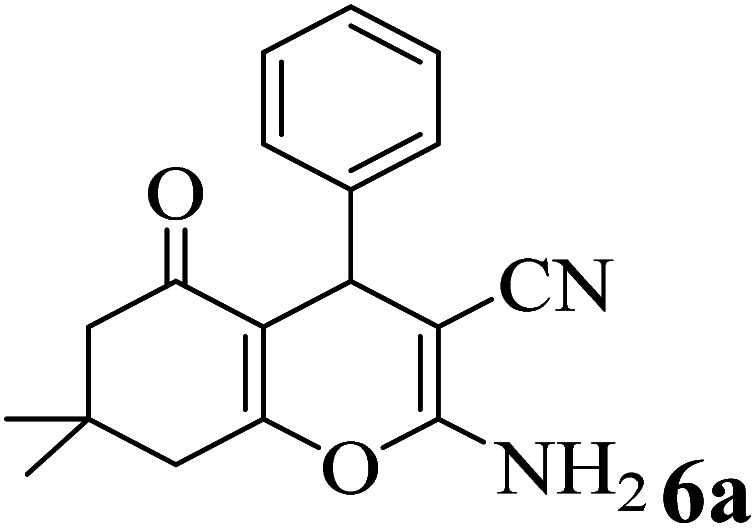
2	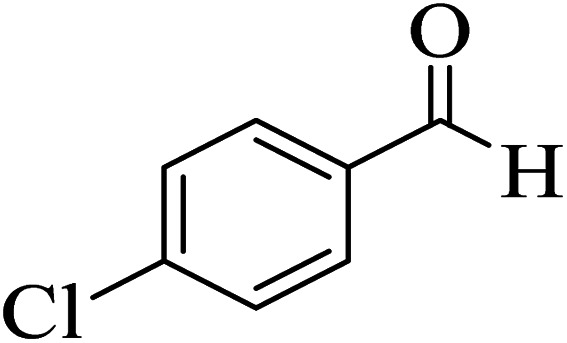	15	96	214–216	212–214 (ref. 54)	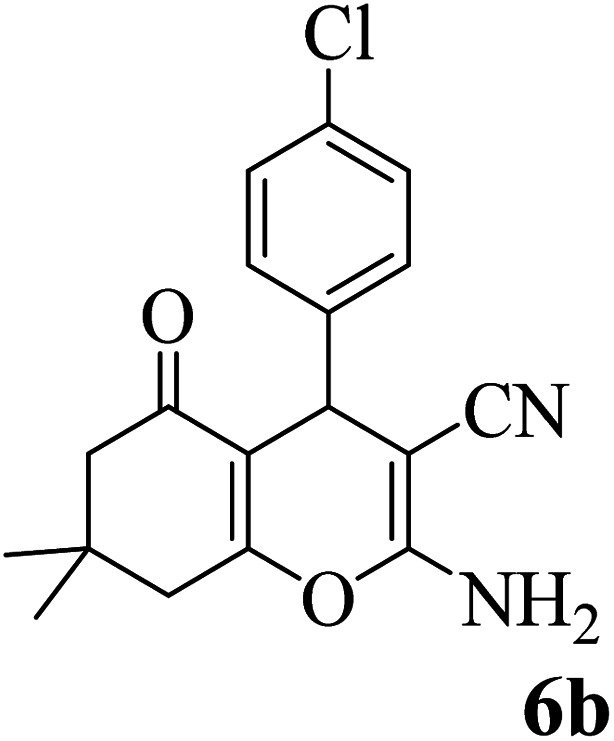
3	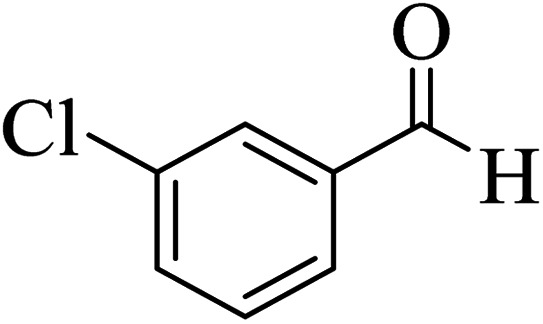	15	92	227–229	228–229 (ref. 55)	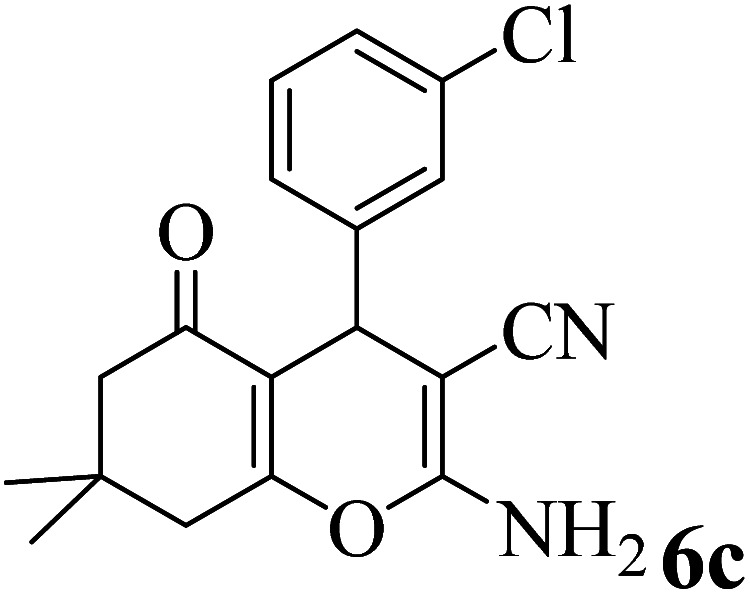
4	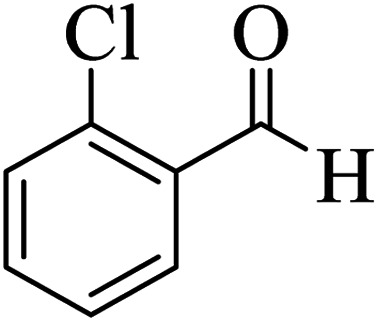	15	95	211–212	210–212 (ref. 56)	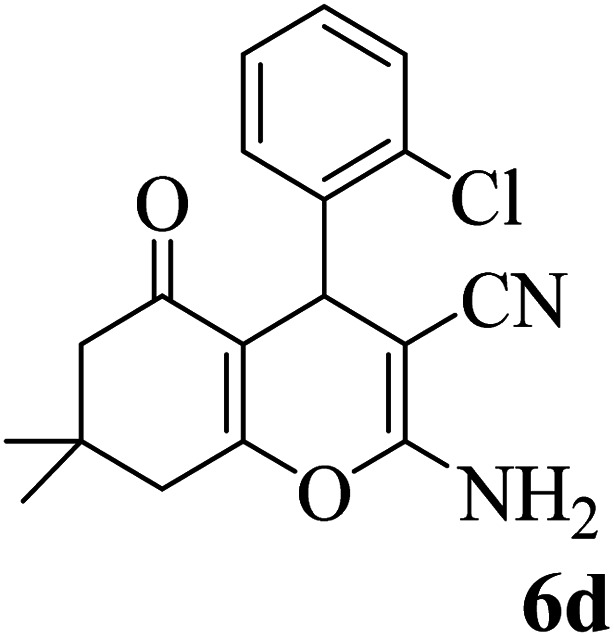
5	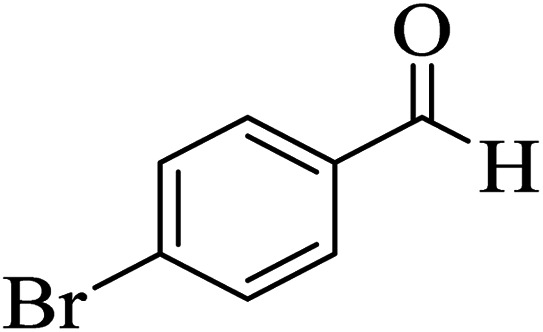	15	92	206–208	207–209 (ref. 57)	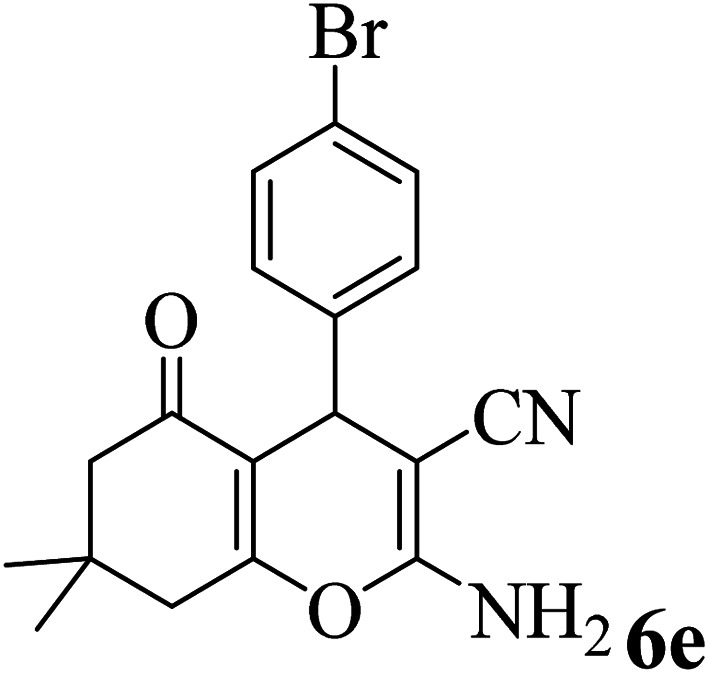
6	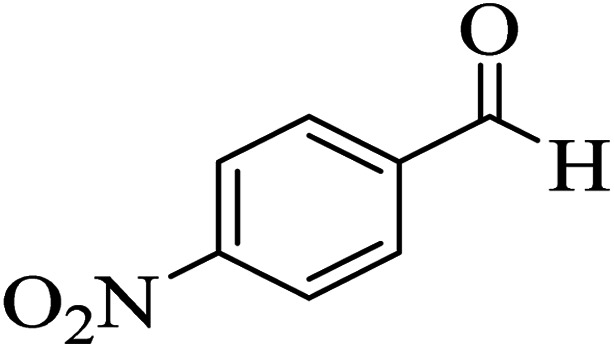	15	92	182–184	178–180 (ref. 58)	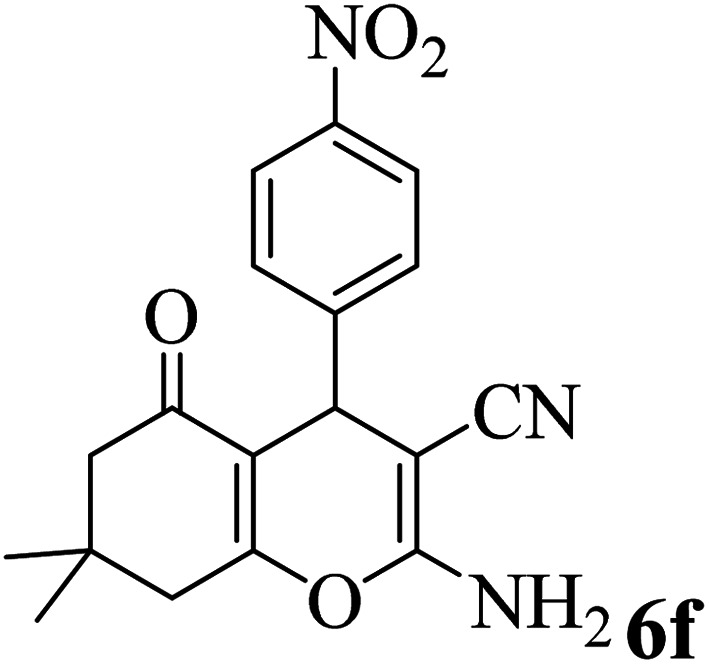
7	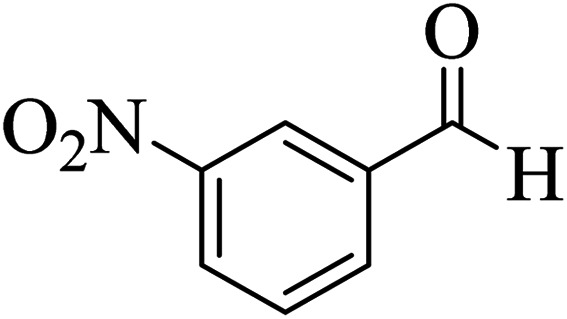	15	94	205–207	208–209 (ref. 58)	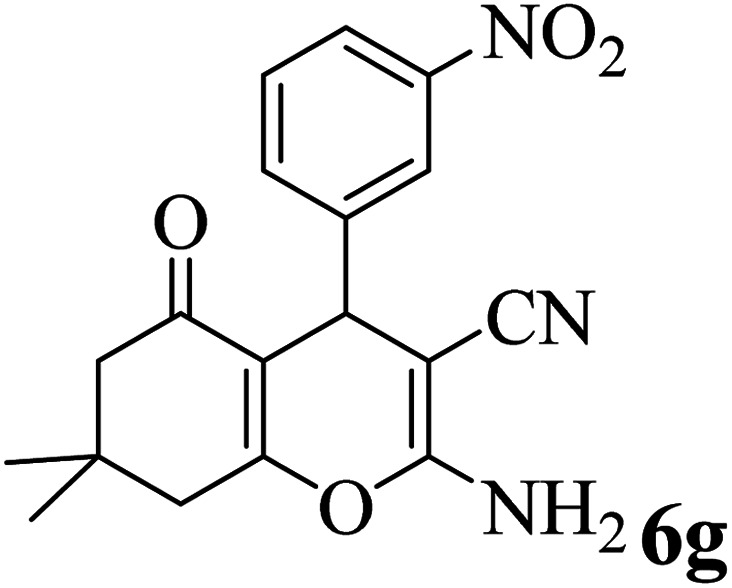
8	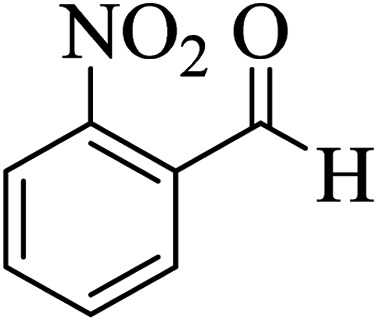	15	95	227–229	228–229 (ref. 58)	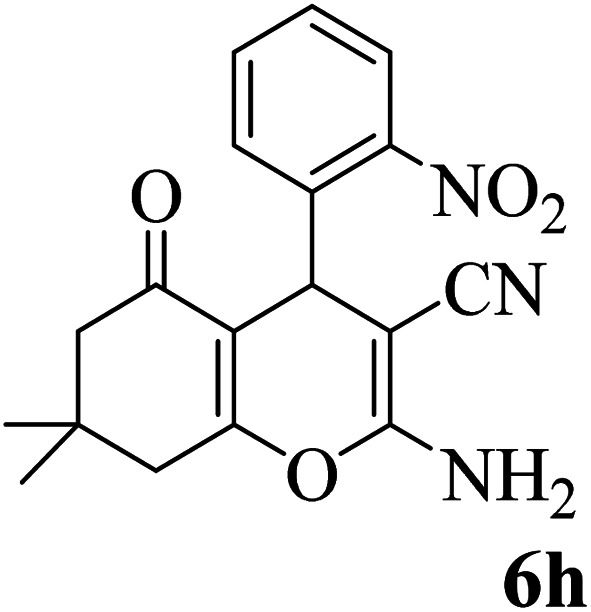
9	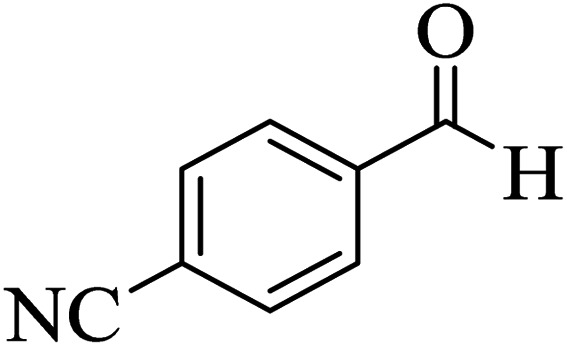	15	92	226–228	227–230 (ref. 57)	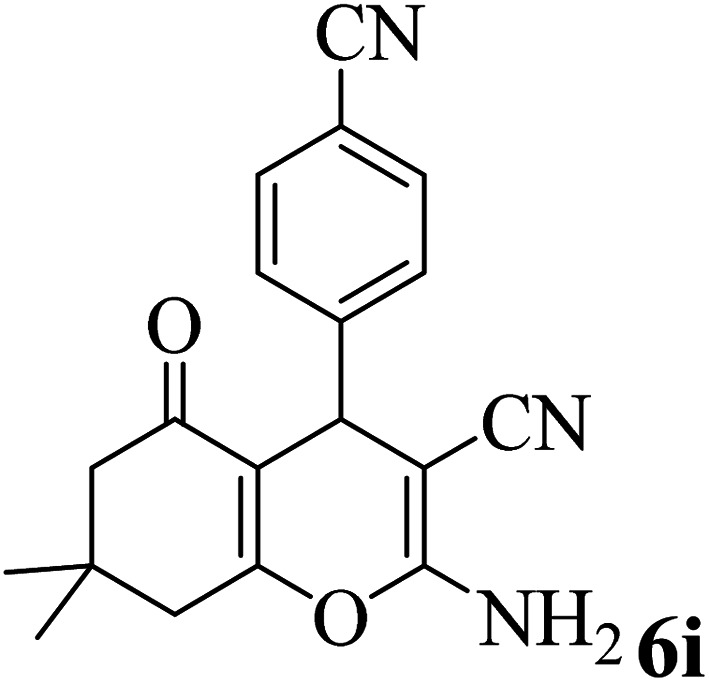
10	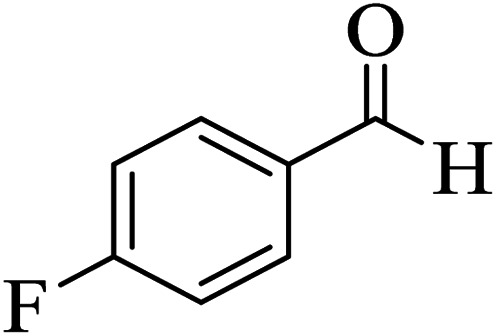	15	95	208–210	210–211 (ref. 59)	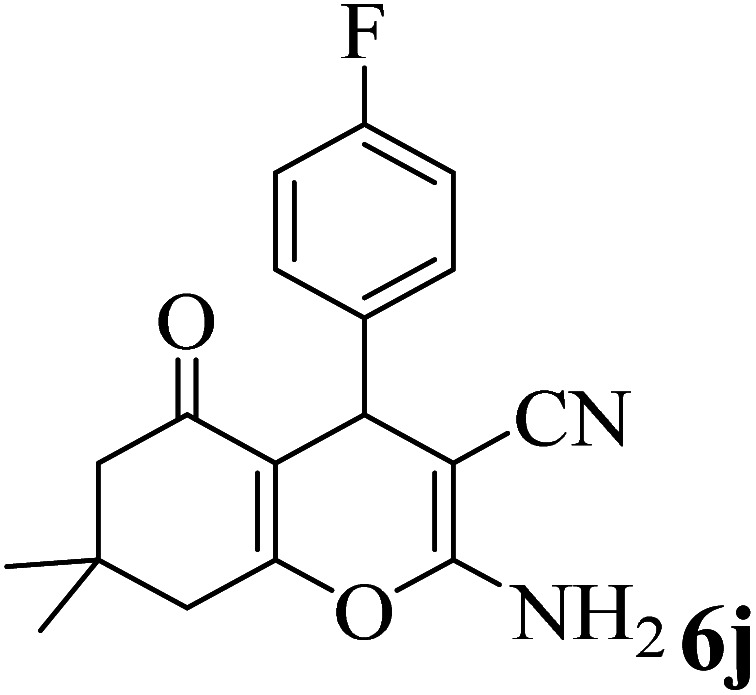
11	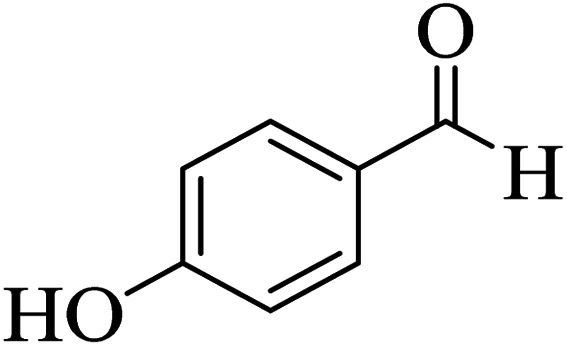	20	92	201–203	203–205 (ref. 58)	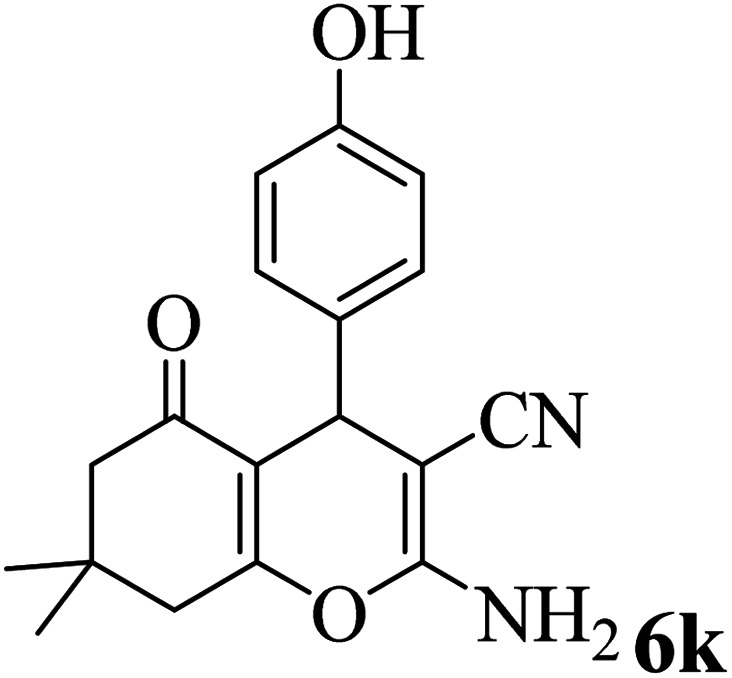
12	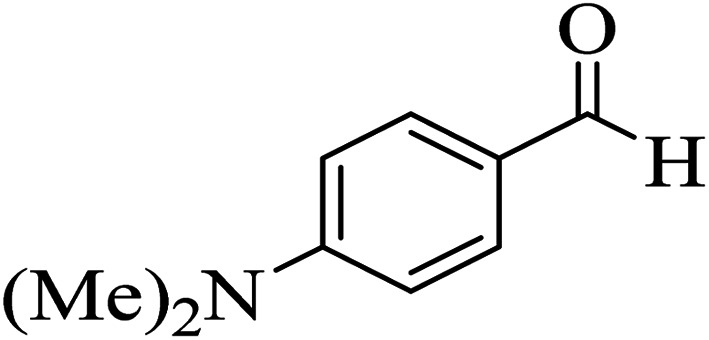	20	92	182–185	174–177 (ref. 45)	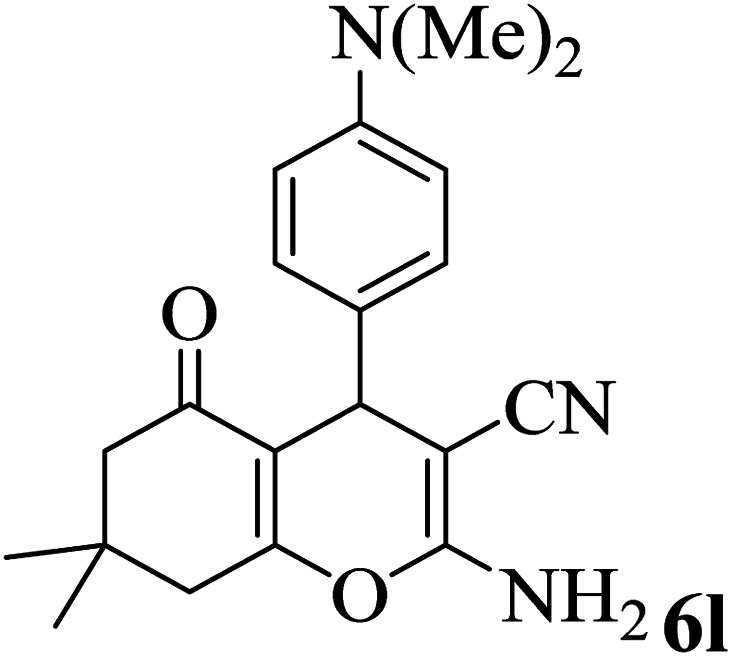
13	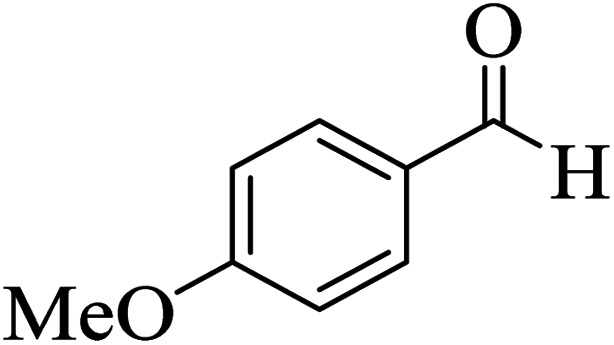	25	90	198–200	194–196 (ref. 60)	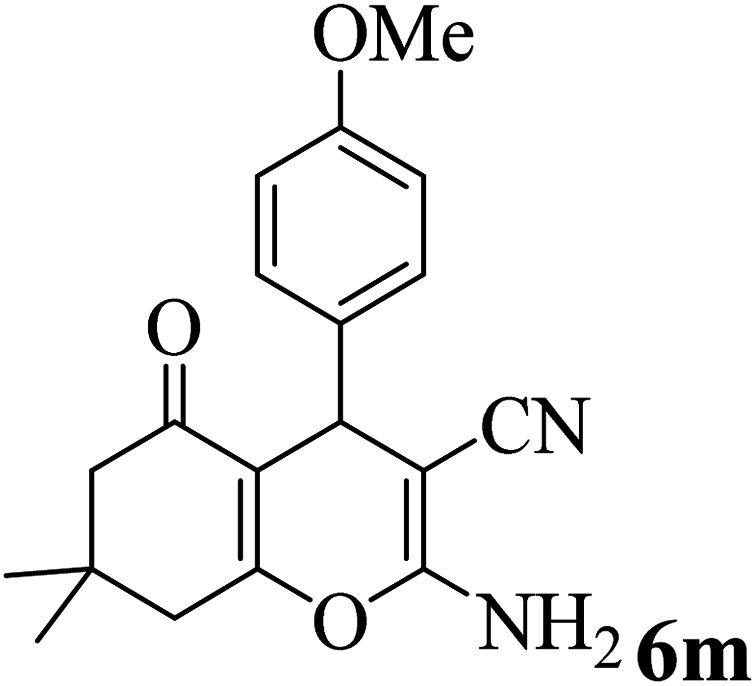
14	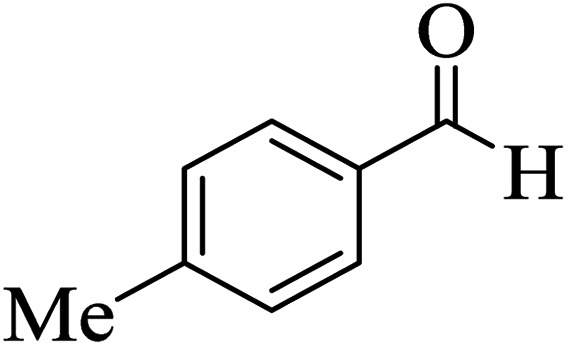	25	91	219–221	223–225 (ref. 61)	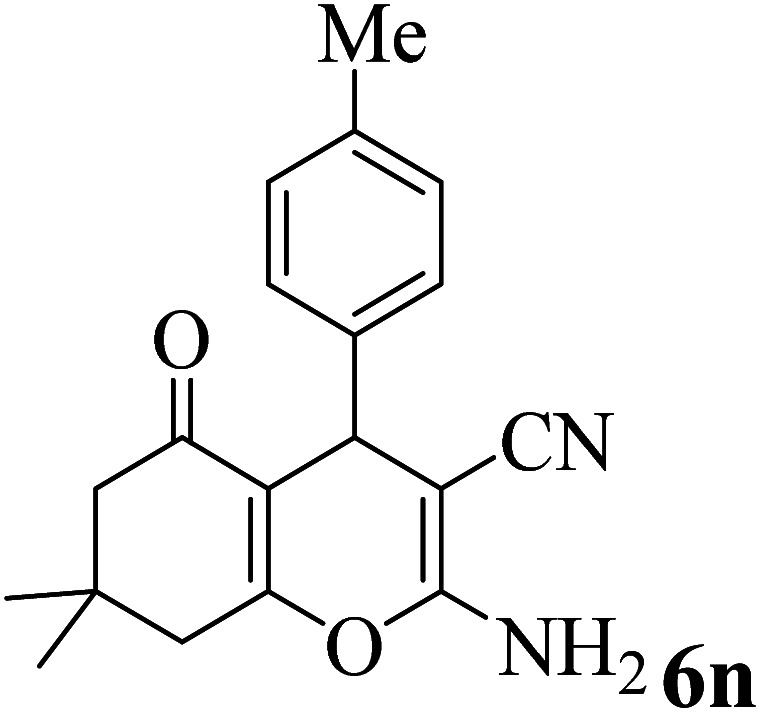

a Reaction conditions: dimedone (1 mmol), malononitrile (1.2 mmol), aldehyde (1 mmol), Zr@IL-Fe_3_O_4_ MNPs (20 mg), solvent-free.

b The yields refer to the isolated product.

**Scheme 3:**
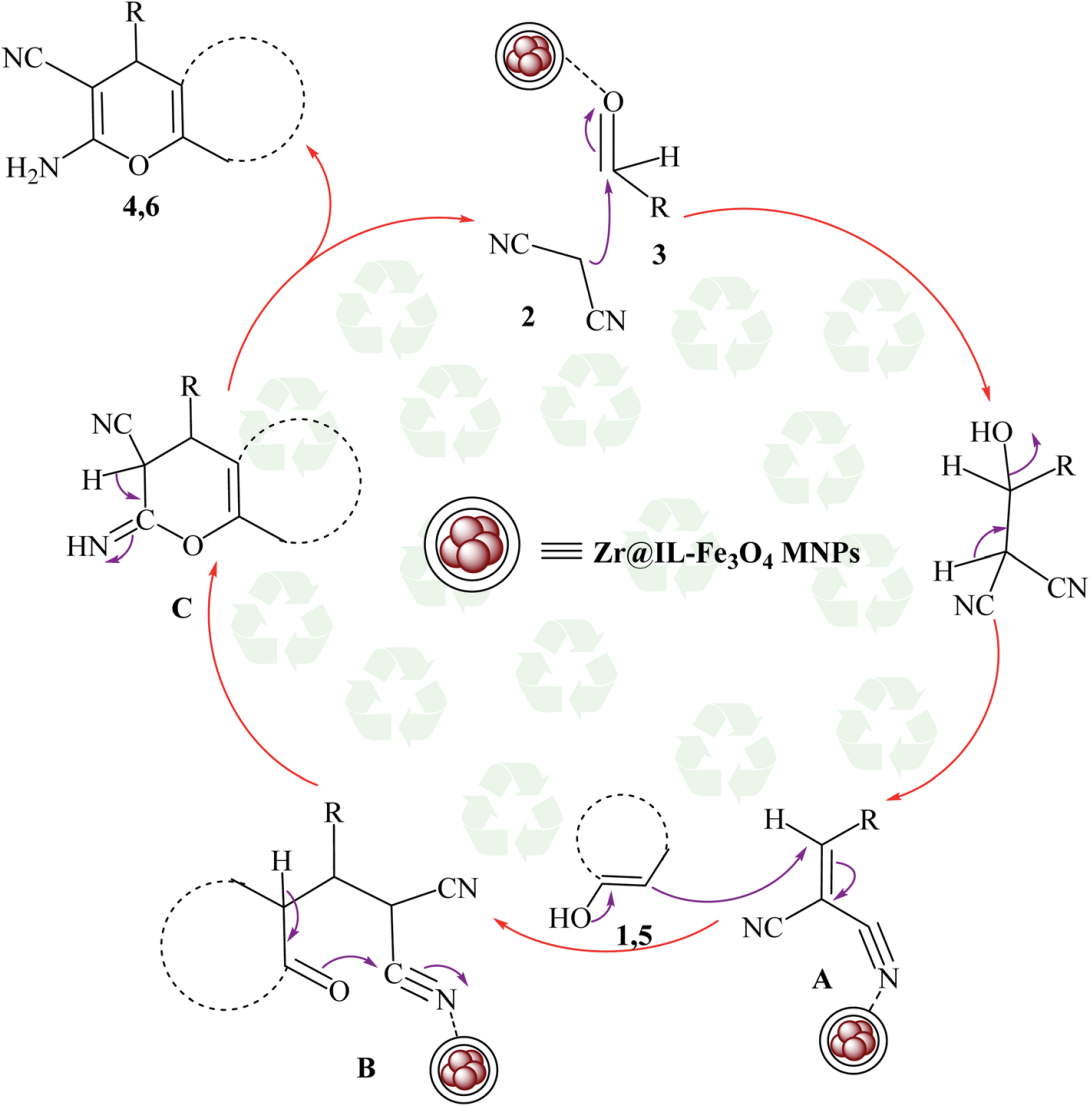
Plausible mechanism for the creation of 2-amino-4*H*-chromene derivatives in the existence of Zr@IL-Fe_3_O_4_ MNPs under solvent-free conditions.

The proposed mechanism for the preparation of the 2-amino-4*H*-chromene derivatives began with the Knoevenagel condensation reaction between malononitrile 2 and aldehyde 3, among which the carbonyl functional group of the aldehyde was activated with the Zr groups of the catalyst. Subsequently, releasing a molecule of water created alkylidene malononitrile (intermediate A). In the next step, intermediate A could be attacked by the Michael addition of the C–H-activated acid (1 and 5) converted to enolate in the presence of the Zr groups of the catalyst. For the preparation of intermediate C from the polar transition state B, a cyclization occurred by the attack of the enolized C–H-activated acid (1 and 5) to the nitrile group. Finally, by an imine-enamine tautomerization of the unstable intermediate C in the presence of the catalyst, the target products were obtained (Scheme 3).

**Fig. 9:**
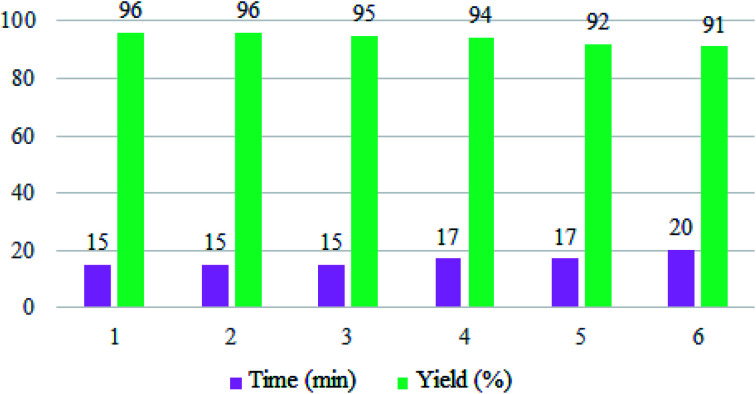
Reusability curves for the preparation of 2-amino-4-(4-chlorophenyl)-5-oxo-4*H*,5*H*-pyrano[3,2-*c*]chromene-3-carbonitrile.

To evaluate the reusability of Zr@IL-Fe_3_O_4_ MNPs, as one of the specified merits of it, a curve of the catalyst performance was prepared for the three-component reaction of 4-hydroxycoumarin, malononitrile, and 4-chlorobenzaldehyde as a model reaction (Fig. 9). At the end of the reaction, a hot mixture of ethyl acetate and ethanol was poured into a flask containing the obtained product. The flask was then placed on a stirrer and after complete dissolution of the product, the reaction solution was decanted with an external magnet into a beaker and the catalyst remained in the reaction vessel. The recovered Zr@IL-Fe_3_O_4_ MNPs were rinsed with ethanol, dried, and reused without a significant reduction in their activity for at least six runs of the reaction.

Table 4 shows the efficiency of the Zr@IL-Fe_3_O_4_ MNPs as a catalyst in the preparation of 2-amino-4-(4-chlorophenyl)-5-oxo-4*H*,5*H*-pyrano[3,2-*c*]chromene-3-carbonitrile compared with some other introduced homogeneous and heterogeneous catalysts. Although all of the mentioned catalysts can partially accelerate the reaction, the present catalytic system had further advantages; for instance, easy work-up procedures, simple recovery of the catalyst, low reaction times, and low catalyst loading.

**Table 4:** Comparison of the results obtained from the synthesis of 2-amino-4-(4-chlorophenyl)-5-oxo-4*H*,5*H*-pyrano[3,2-*c*]chromene-3-carbonitrile using Zr@IL-Fe_3_O_4_ MNPs with other reported strategies^a^

Entry	Catalyst	Solvent	Time (min)	Yield (%)	Ref.
1	SDS	20 mol%/H_2_O/60 °C	150	88	47
2	MNP@AVOPc	20 mg/solvent-free/25 °C	15	94	62
3	[Sipim]HSO_4_	0.1 mmol/solvent-free/100 °C	30	95	63
4	Nano Al_2_O_3_	25 mol%/EtOH/25 °C	300	80	64
5	t-ZrO_2_ NPs	10 mol%/H_2_O/80 °C	35	91	65
6	DMAP	20 mol%/EtOH/reflux	300	94	66
7	DTP/SiO_2_	20 mol%/DMF/60 °C	35	92	67
8	MgO	0.5 mmol%/H_2_O/reflux	120	84	68
9	Zr@IL-Fe_3_O_4_ MNPs	20 mg/solvent-free/100 °C	15	96	This work

a Reaction conditions: 4-hydroxycoumarin (1 mmol), malononitrile (1.2 mmol), 4-chlorobenzaldehyde (1 mmol).

## Conclusion

In summary, we described an effective procedure for the synthesis of highly substituted pyran derivatives *via* a one-pot three-component condensation of 4-hydroxycoumarin (1)/dimedone (5), malononitrile (2), and arylaldehydes (3) using a Zr@IL-Fe_3_O_4_ MNP heterogeneous magnetic nanocatalyst under solvent-free conditions. The catalyst was characterized *via* Fourier transform infrared (FT-IR) spectroscopy, energy dispersive X-ray spectroscopy (EDX), vibrating sample magnetometry (VSM), X-ray diffraction (XRD), thermogravimetric analysis (TGA), transmission electron microscopy (TEM), and scanning electron microscopy (SEM) techniques. The important features of the suggested strategy include a high efficiency of the catalyst, reusability of the catalyst through the use of an external magnetic field, high-to-excellent yields of the products, and short reaction times.

## Conflicts of interest

There are no conflicts to declare.

## Supplementary Material
